# Recombinant dermatan sulfate is a potent activator of heparin cofactor II-dependent inhibition of thrombin

**DOI:** 10.1093/glycob/cwz019

**Published:** 2019-04-23

**Authors:** Emil Tykesson, Marco Maccarana, Hanna Thorsson, Jian Liu, Anders Malmström, Ulf Ellervik, Gunilla Westergren-Thorsson

**Affiliations:** 1 Department of Experimental Medical Science, BMC C12, Lund University, Lund, Sweden; 2 Division of Chemical Biology and Medicinal Chemistry, Eshelman School of Pharmacy, University of North Carolina, Rm 303, Beard Hall, Chapel Hill, NC, USA; 3 Department of Chemistry, Lund University, Box 124, Lund, Sweden

**Keywords:** coagulation, dermatan sulfate, glycosaminoglycans, heparin cofactor II

## Abstract

The glycosaminoglycan dermatan sulfate (DS) is a well-known activator of heparin cofactor II-dependent inactivation of thrombin. In contrast to heparin, dermatan sulfate has never been prepared recombinantly from material of non-animal origin. Here we report on the enzymatic synthesis of structurally well-defined DS with high anticoagulant activity. Using a microbial K4 polysaccharide and the recombinant enzymes DS-epimerase 1, dermatan 4-*O*-sulfotransferase 1, uronyl 2-*O*-sulfotransferase and *N*-acetylgalactosamine 4-sulfate 6-*O*-sulfotransferase, several new glycostructures have been prepared, such as a homogenously sulfated IdoA-GalNAc-4S polymer and its 2-*O*-, 6-*O*- and 2,6-*O*-sulfated derivatives. Importantly, the recombinant highly 2,4-*O*-sulfated DS inhibits thrombin via heparin cofactor II, approximately 20 times better than heparin, enabling manipulation of vascular and extravascular coagulation. The potential of this method can be extended to preparation of specific structures that are of importance for binding and activation of cytokines, and control of inflammation and metastasis, involving extravasation and migration.

## Introduction

Antithrombin (ATIII) and heparin cofactor II (HCII) are the serpins responsible for in vivo inhibition of thrombin (Tollefsen et al. 1983). Upon interaction with heparin or dermatan sulfate (DS), the rate of inhibition is increased 1000-fold (Jordan et al. 1980; Tollefsen et al. 1982). Whereas heparin affects thrombin inhibition via both ATIII and HCII, DS acts solely through HCII in the vessel walls after endothelial damage (Figure 2A) (He et al. 2008). DS is composed of alternating *N*-acetyl-d-galactosamine (GalNAc) and l-iduronic acid (IdoA) residues. To form IdoA in DS, two chain-modifying enzymes are required; DS epimerase 1 (DS-epi1) and dermatan 4-*O*-sulfotransferase 1 (D4ST1) (Evers et al. 2001; Maccarana et al. 2006). DS-epi1 is responsible for the inversion of stereochemistry of carbon 5 in GlcA, to form IdoA, while D4ST1 transfers a sulfate group from 3′-phosphoadenosine 5′-phosphosulfate (PAPS) to C4 of a GalNAc adjacent to IdoA (Evers et al. 2001). In addition, uronyl 2-*O*-sulfotransferase (UST) and *N*-acetylgalactosamine 4-sulfate 6-*O*-sulfotransferase (GalNAc4S-6ST) transfer sulfate groups to C2 of IdoA and C6 of GalNAc4S, respectively, generating di- and trisulfated structures. DS (MF 701) has been used as an anticoagulant in hemodialysis, disseminated intravascular coagulation and hip fractures (Cohen et al. 1994). Furthermore, danaparoid, a combination of heparan sulfate and DS, is used in heparin-induced thrombocytopenia (Acostamadiedo et al. 2005). These glycosaminoglycan-derived anticoagulants are far from ideal drugs due to their (i) animal origin, (ii) narrow therapeutic window and (iii) inherent structural dishomogeneity. The latter problems were exposed in the 2008 heparin crisis where oversulfated chondroitin sulfate illegally added to heparin caused fatal anaphylactic reactions (Guerrini et al. 2008). To address some of these problems, we initiated a study to produce structurally well-defined recombinant DS (recDS) with anticoagulant activity from the microbial K4 polysaccharide, using the recombinant enzymes DS-epi1, D4ST1, UST and GalNAc4S-6ST.

## Results and discussion

### Structurally well-defined recombinant dermatan sulfate can be produced from a bacterial polysaccharide substrate

**Fig. 1.**
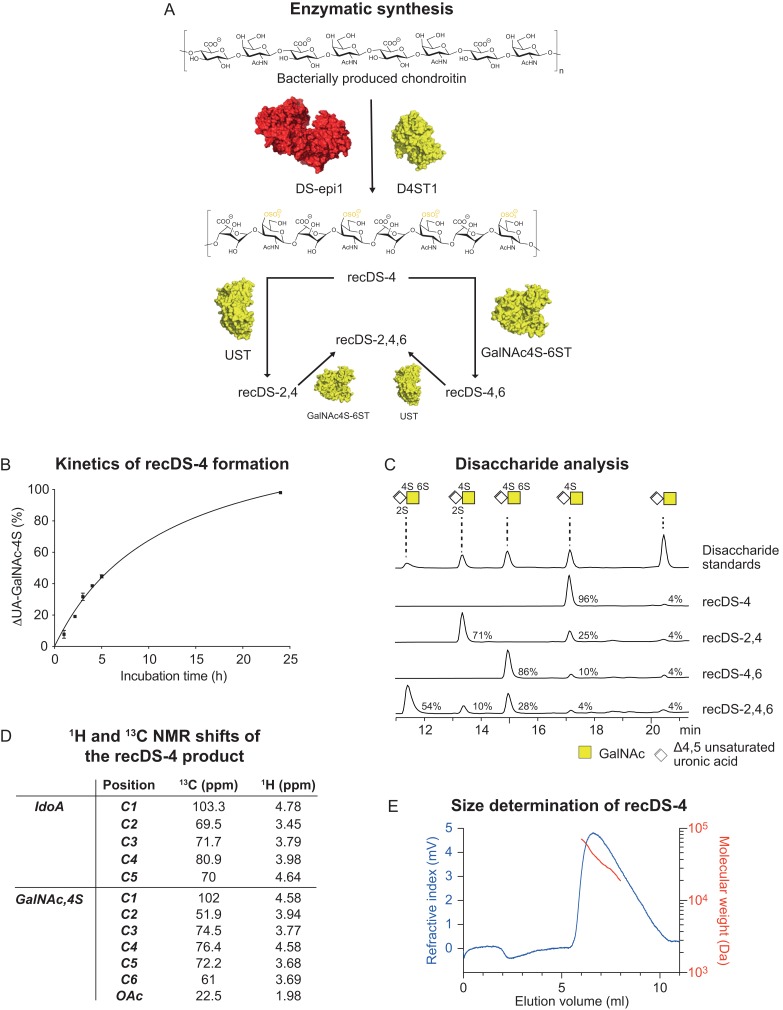
Synthesis and analysis of recombinant anticoagulant DS. (**A**) Synthesis scheme for recDS production. (**B**) Kinetics of recDS-4 formation. By varying the enzymatic incubation time, the degree of modification can be tuned. Two repeats were performed, both in triplicates, giving similar results. Representative data from one repeat is shown as the mean ± one standard deviation. (**C**) Disaccharide analysis of the recDS products after chondroitinase ABC digestion. (**D**) ^1^H and ^13^C NMR shifts of the recDS-4 product. (**E**) Size analysis of the recDS-4 product by multi-detection size exclusion chromatography, showing the refractive index signal in blue and the absolute molecular weight around the peak apex in red.

As previously shown, incubation of chondroitin with DS-epi1 alone results in short stretches of IdoA-containing sequences (Tykesson et al. 2016). In order to produce long IdoA-blocks (Tykesson et al. 2018), DS-epi1 and D4ST1 were co-incubated with chondroitin and PAPS for 24 h (Figure 1A and B) to give a product (recDS-4) containing 96% UA-GalNAc-4S, determined by disaccharide analysis after chondroitinase ABC (Figure 1C). The presence of IdoA and GalNAc-4S was shown by disaccharide analysis after chondroitinase B (data not shown) and confirmed by NMR spectroscopy (Figure 1D). Further characterization by SEC-MALS showed a weight average molecular weight (*M*_w_) of 35.5 kDa with a dispersity of 1.14 (Figure 1E). Based on the sulfation degree and the size of the polymers it can be concluded that the 4% of non-sulfated disaccharide structures in recDS-4 are most likely positioned on the reducing and non-reducing ends of the polymer, in agreement with the substrate specificity of both DS-epi1 and D4ST1 (Evers et al. 2001; Mikami et al. 2003; Tykesson et al. 2016). Depending on the potential future applications of the recDS, we could also show that it is also possible to tune the degree of 4-*O*-sulfated residues by varying the enzymatic incubation time (Figure 1B). In contrast to the heterogeneity of commercial 4-*O*-sulfated DS preparations isolated from animal sources, the unique recDS-4 preparation is homogenously composed of IdoA-GalNAc-4S along the polymer and can be used to evaluate the biological properties and functions of 4-*O*-sulfated DS.

The recDS-4 product was further sulfated using UST to form a 71% 2,4-*O*-sulfated recDS-2,4 (Figure 1C). Interestingly, initial experiments showed that 2-*O*-sulfation of unsulfated chondroitin or dermatan was slow and few sulfate groups were added. We propose that UST preferably transfers sulfate groups to 4-*O*-sulfated DS, in agreement with earlier observations (Kobayashi et al. 1999). Finally, disulfated 4,6-*O*- (86%) or trisulfated 2,4,6-*O*- (54%) recDS could be obtained by incubation of recDS-4 or recDS-2,4, respectively, with GalNAc4S-6ST (Figure 1C). These results extend the substrate specificity of GalNAc4S-6ST to 2,4-*O*-sulfated DS, in addition to the previously known mono-4-*O*-sulfated substrates (Ohtake et al. 2001).

### A recombinant 2,4-*O*-sulfated DS is a potent activator of HCII-dependent inhibition of thrombin

**Fig. 2.**
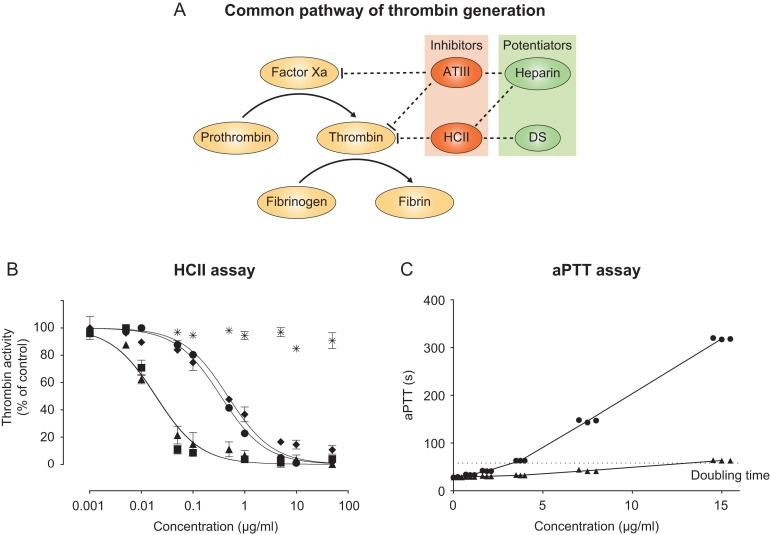
Evaluation of the anticoagulant properties of recombinant DS. (**A**) Simplified overview of the common pathway of thrombin generation. Heparin inhibits coagulation via both ATIII and HCII, whereas DS has a singular anticoagulant activity, via HCII. (**B**) Inhibition of thrombin by HCII in the presence of heparin (●), recDS-4 (✳), recDS-4,6 (♦), recDS-2,4 (▲), and recDS-2,4,6 (■). Two repeats were performed, both in triplicates, giving similar results. Representative data from one repeat is shown as the mean ± one standard deviation. (**C**) Activated partial thromboplastin time of heparin (●) and recDS-2,4 (▲) at various concentrations. Replicate data points are shown staggered for clarity. Results are from one experiment performed in triplicates using the SWEDAC accredited method (NPU01682) at the Skåne University Hospital.

To investigate the functionality of our recDS preparations, an assay for the HCII-dependent inactivation of thrombin was set up. The 4-*O*-sulfated recDS showed no activity. However, the 4,6-*O*-sulfated recDS with 86% IdoA-GalNAc-4,6 sulfate exhibited a HCII-dependent inactivation of thrombin with similar potency as heparin, i.e., IC_50_ values of 479 ± 95 ng/mL and 348 ± 25 ng/mL, respectively (Figure 2B). A DS preparation with 9% 4,6-*O*-sulfated structures has previously been shown to weakly inhibit thrombin via HCII, suggesting that a significantly higher proportion of disulfated structures is necessary for efficient inhibition (Halldórsdóttir et al. 2006). Most importantly, we found that a preparation with 71% of IdoA-2S-GalNAc-4S had an IC_50_ value of 19 ± 4 ng/mL, i.e., 15-20 times better inhibition compared to heparin (Maimone and Tollefsen 1990). To our knowledge, this is the most potent native polysaccharide activator of HCII to date. Trisulfated recDS had an IC_50_ value of 19 ± 6 ng/mL, suggesting that additional sulfation does not improve activation.

In order to verify these results in in vivo-like settings, we analyzed the ability of the 2,4-*O*-sulfated recDS to prolong the activated partial thromboplastin time (aPTT) (Figure 2C). The concentration required to double the aPTT was 13.0 ± 0.8 μg/mL for 2,4-*O*-sulfated recDS, compared to 3.5 ± 0.2 μg/mL for heparin. Even though the 2,4-*O*-sulfated recDS only acts via HCII, and not ATIII (Figure 2A), the activity is four times lower than that of heparin when comparing mass concentrations and only one point five times lower when comparing molar concentrations (~16 kDa versus ~40 kDa for heparin and recDS-2,4, respectively).

While chemoenzymatic synthesis is highly developed for heparin/heparan sulfate, the production of recombinant dermatan sulfate has so far gained less attraction (Xu et al. 2011; Zhang et al. 2017). In this article we report that functional DS, of non-eukaryotic origin, with well-defined chemical structures can be produced, enabling production of DS with different amounts of IdoA, 2-*O*-, 4-*O*-, and 6-*O*-sulfate.

The control of blood coagulation is of outermost importance, and we show that recombinant DS inhibits thrombin via heparin cofactor II, approximately 20 times better than heparin, enabling manipulation of vascular and extravascular coagulation. Based on previously reported functions of mammalian DS, our method can potentially be extended to preparation of structures that are of importance for binding and activation of cytokines, control of collagen matrix structure, inflammation and metastasis involving P-selectin mediated extravasation and cancer cell migration (Maccarana et al. 2009; Kozlowski et al. 2011; Thelin et al. 2012; Mizumoto et al. 2013; Westergren-Thorsson et al. 2016).

## Materials and methods

### Materials

PAPS and chondroitin was prepared as described previously (Hannesson et al. 1996; Zhou et al. 2011). Unfractionated heparin sodium salt with a weight average molecular weight (*M*_w_) of 15.5 kDa and a dispersity of 1.28 (determined by multi-detection SEC, as below) was from porcine intestinal mucosa (Sigma-Aldrich H3393, Grade I-A, 179 USP units/mg).

### Cloning and expression of DS-epi1, D4ST1, UST and GalNAc4S-6ST

DS-epi1 and D4ST1 were cloned and expressed as previously described (Tykesson et al. 2016, 2018).

The part of the open reading frame of the human uronyl 2-*O*-sulfotransferase (UST) gene *UST* (sequence harmonized, Genewiz, USA) corresponding to the lumenal amino acids 71 to 406 was subcloned together with a C-terminal 8xHIS tag into the NheI and NotI sites of a pCEP-Pu/BM40 (Kohfeldt et al. 1997) (modified version of pCEP4 from Invitrogen) expression vector using the following primers (Sigma-Aldrich):

Forward (NheI restriction site in bold letters)

Amino acid 71:5′-GCATCT**GCTAGC**CCCCCCTAGATTCCTGCTCG-3′

Reverse (NotI restriction site in bold letters)

Amino acid 406: 5′-GCATCT**GCGGCCGC**TCAATGGTGATGGTGATGATGGTGGTGTCTTTTGTAGATGTCCTCGAGC-3′.

The part of the open reading frame of the human *N*-acetylgalactosamine 4-sulfate 6-*O*-sulfotransferase (GalNAc4S-6ST) gene *CHST15* (sequence harmonized, Genewiz, USA) corresponding to the lumenal amino acids 102 to 561 was subcloned together with a C-terminal 8xHIS tag into the NheI and NotI sites of pCEP-Pu/BM40 using the following primers (Sigma-Aldrich):

Forward (NheI restriction site in bold letters)

Amino acid 102: 5′-GCATCT**GCTAGC**CCATCAGGAGCTCCTGATTTCC -3′

Reverse (NotI restriction site in bold letters)

Amino acid 561: 5′-GCATCT**GCGGCCGC**TCAATGGTGATGGTGATGATGGTGGTGGGTGGTCTTCCAAGCGAAAG-3′

Transfection, expression and purification was performed as previously described (Tykesson et al. 2016).

### Preparation of recombinant dermatan sulfate

**recDS-4:** DS-epi1 (242 μg, 2.7 nmol) was mixed with D4ST1 (266 μg, 5.9 nmol), PAPS (33 μmol), bovine serum albumin (BSA) (120 mg) and chondroitin (308 μg, 0.81 μmol HexA) in final 100 mL MES buffer (20 mM, pH 6.5) supplemented with MnCl_2_ (10 mM). The sample was incubated at 37°C for 24 h under agitation. The polysaccharide product was purified by anion exchange chromatography on a 1 mL HiTrap DEAE FF column (GE Healthcare) equilibrated with 50 mM NaOAc, pH 5.0 at 1 mL/min, using an ÄKTA Start (GE Healthcare). The DEAE column was then washed with 5 mL 50 mM NaOAc, pH 5.0 and finally eluted with a 30 mL gradient, going from 50 mM NaOAc, pH 5.0, to 50 mM NaOAc, pH 5.0, +2 M NaCl. Fractions of interest were desalted with milli-Q water on a 4 mL Amicon Ultra 10 kDa column (Millipore).

**recDS-2,4:** UST (1700 μg, 32.1 nmol) was mixed together with PAPS (17.5 μmol), BSA (50 mg) and 4-*O*-sulfated recombinant DS (115 μg, 0.25 μmol HexA) in final 50 ml MES buffer (20 mM, pH 6.5) supplemented with MnCl_2_ (10 mM). The sample was incubated at 37°C for 48 h under agitation. The polysaccharide product was purified as above.

**recDS-4,6:** GalNAc4S-6ST (12.5 μg, 0.2 nmol) was mixed together with PAPS (17.5 μmol), BSA (50 mg) and 4-*O*-sulfated recombinant DS (115 μg, 0.25 μmol HexA) in final 50 mL MES buffer (20 mM, pH 6.5) supplemented with MnCl_2_ (10 mM). The sample was incubated at 37°C for 48 h under agitation. The polysaccharide product was purified as above.

**recDS-2,4,6:** UST (120 μg, 2.3 nmol) was mixed together with PAPS (50 μL, 1.75 μmol), BSA (5 mg) and 4,6-*O*-sulfated recombinant DS (11 μg, 20 nmol HexA) in final 5 mL MES buffer (20 mM, pH 6.5) supplemented with MnCl_2_ (10 mM). The sample was incubated at 37°C for 24 h in an Eppendorf ThermoMixer C operated with vortexing at 600 rpm. The polysaccharide product was purified as above.

### Disaccharide analysis

Disaccharide analysis was essentially performed as described previously (Stachtea et al. 2015). In short, samples were buffer exchanged into an ammonium acetate buffer (50 mM, pH 7.5) and to each sample, in approximately 30 μL, chondroitinase ABC (10 mIU, Sigma-Aldrich) was added to depolymerize the polysaccharide products to Δ^4,5^-unsaturated uronic acid containing disaccharides. Depolymerization was achieved by incubation at 37°C for 4 h, after which the samples were boiled, centrifuged at 20,000 × *g* for 10 min and the supernatant was dried in a centrifugal concentrator and saved for future analysis. Pre-column, 2-aminoacridone-labeled DS disaccharides were analyzed on a Thermo Scientific UltiMate 3000 Quaternary Analytical system equipped with an FLD-3400RS fluorescence detector. For recDS-4, the polysaccharides were also degraded using chondroitinase B (2 mIU, R&D Systems) in 30 μL ammonium acetate buffer (50 mM, pH 7.5) overnight at 37°C. Disaccharide standards were from Iduron (Manchester, UK).

### Multi-detection SEC

SEC was performed using a Malvern Panalytical OMNISEC system (Malvern, UK) consisting of Refractive Index (RI), Right Angle and Low Angle light scattering (RALS/LALS) and differential viscometer. All data was collected and processed using OMNISEC v10. For chromatographic separation, a Malvern Panalytical PLS3030 column (300 Å, 3 μM, 7.8 × 300 mm) was used with PBS buffer. For analysis of the polysaccharides a dn/dc of 0.12 ml/g was used as it was assumed that all samples had a composition like that of heparin.

### NMR analysis

Experiments were performed at 298 K on a Bruker Avance III HD 800 MHz equipped with a TXO cryo probe and referenced to HDO at 4.70 ppm, with the following settings: **1 H 1D.** Pulse sequence zg30, 256 scans, acquisition time 2.05 s. Alternatively, pulse sequence 1D with excitation sculpting with perfect echo based on zgespe, 512 scans, acquisition time 2.05 s. **13 C 1D.** Pulse sequence zgzrse, 32768 scans, acquisition time 1.024 s. **1H-13C HSQC.** Pulse sequence hsqcedetgpsisp2.4, 64 scans, acquisition time 80 ms, TD 256 × 2048 (f1 × f2), dummy scans 32, offset 13 C 80.0 ppm. Spectra were analyzed using TopSpin 3.5pl7.

### Inhibition of thrombin by heparin cofactor II and DS

Glycosaminoglycans (0–50 μg/mL, quantified by disaccharide analysis) were mixed with human thrombin (1 nM, R&D Systems) in 15 μL buffer composed of Tris-HCl (50 mM, pH 7.5), sodium chloride (150 mM), calcium chloride (10 mM) and Brij-35 (0.05% (v/v)). The mixture was incubated for 5 min at 22°C before addition of HCII (15 μL, 30 nM, R&D Systems). After 15 min at 22°C, Chromozym TH (100 μL, 0.2 mM, Roche) was added and the absorbance measured at 405 nm for 600 s at 20 s intervals. All the above concentrations in parenthesis are final. All experiments were performed in triplicates.

### aPTT analysis

aPTT was measured in triplicates by a SWEDAC accredited (ISO 15189) medical laboratory at the Skåne University Hospital on a CS-5100 automated analyzer (Siemens, Marburg, Germany) using the Actin FSL reagent (Siemens). The reference interval was 26-33 s with a coefficient of variation of <4%.

### Statistical analysis

Data are expressed as mean values ± one standard deviation of experiments performed in triplicates, calculated using GraphPad Prism version 8.0.0.
